# The Approximate Number System and Mathematical Abilities in Chinese Preschoolers With and Without Autism Spectrum Disorder

**DOI:** 10.3390/jintelligence14040071

**Published:** 2026-04-21

**Authors:** Lilan Chen, Zhiyong Zhong, Wenyuan Jiang

**Affiliations:** School of Psychology, Hainan Normal University, Haikou 571158, China

**Keywords:** mathematical abilities, approximate number system, formal and informal mathematics, preschoolers, cross-sectional study

## Abstract

Mathematical abilities are critical for the developmental outcomes of children with autism spectrum disorder (ASD). However, little is known about these abilities and their association with the approximate number system (ANS) in preschoolers with ASD beyond Western samples, including Chinese children. This cross-sectional study examined whether formal and informal mathematical abilities differed between children with and without ASD and assessed the extent to which these abilities were associated with ANS acuity. Participants included 47 children with ASD and 47 typically developing (TD) children aged 3–7 years. All children were assessed on measures of formal and informal mathematical abilities, ANS acuity, and non-verbal IQ. No significant group differences in mathematical abilities were found among children aged 3–5 years. However, among children aged 6–7 years, the ASD group showed significantly lower performance in mathematical abilities compared to their TD peers. ANS acuity was significantly correlated with both formal and informal mathematical abilities in the ASD group, but only with informal mathematical abilities in the TD group. Furthermore, ANS acuity accounted for 5.4% of the unique variance in formal mathematical abilities specifically within the ASD group. The patterns of mathematical abilities and their relationship with ANS acuity differ between preschoolers with and without ASD. These findings suggest a differential association between ANS and formal mathematics learning in children with ASD, highlighting implications for the design of early numeracy interventions.

## 1. Introduction

With the advancement of inclusive education, a growing number of children with autism spectrum disorder (ASD) are enrolled in general education classrooms, where they receive formal instruction alongside their typically developing peers (McDonald et al., 2019). This shift has heightened interest in the academic achievement of children with ASD, particularly in the domain of mathematics. Mathematical ability is often categorized into informal and formal competencies. According to Ginsburg and Baroody (1990), informal mathematical ability refers to concepts and skills acquired outside of school settings, reflecting an implicit understanding of number and quantity, whereas formal mathematical ability involves arithmetic and conceptual knowledge explicitly taught in school. For example, informal mathematical abilities include counting objects, sharing snacks equally, and comparing which of two groups has more items; formal mathematical abilities include solving written arithmetic problems (e.g., 3 + 2 = ?), recognizing number symbols, and understanding place value. Informal mathematical ability is considered foundational to the development of formal mathematical skills (Raman, 2002). A substantial body of literature has highlighted the role of parents in facilitating informal mathematical learning, such as counting objects, playing number games, and engaging in everyday numerical activities (Susperreguy & Davis-Kean, 2016; Zippert et al., 2019). However, the development of these abilities in preschoolers with ASD remains underexplored. Children with ASD show significant heterogeneity in cognitive and behavioral profiles. Moreover, little is known about this topic in non-Western educational contexts such as China.

The approximate number system (ANS) supports the rapid estimation of quantities without counting. It is regarded as one of the core foundational capacities underlying numerical cognition (Feigenson et al., 2004). Present from infancy in humans and shared across many species (Dehaene, 2011), the ANS is thought to support the acquisition of symbolic numerical knowledge and the growth of more complex mathematical abilities (Feigenson et al., 2013; Mussolin et al., 2016). Research interest in the ANS has continued to grow in recent years, with studies extending its investigation to diverse populations and developmental contexts (L. Wang et al., 2023; X. Li et al., 2024). While a substantial body of research has established correlational links between ANS acuity and mathematical achievement in typically developing children, evidence for this association in children with ASD remains limited and largely cross-sectional.

### 1.1. Mathematical Abilities of Children with ASD

Research findings on the mathematical abilities of individuals with autism spectrum disorder (ASD) have been notably inconsistent. To understand these discrepancies, we organize prior findings into three theoretical perspectives: (a) cognitive strengths versus deficits, (b) the role of IQ, and (c) heterogeneity within the ASD population.

From a cognitive strengths versus deficits perspective, some studies suggest that mathematics may be an area of relative strength for individuals with ASD (Baron-Cohen et al., 2007; Jones et al., 2009; Wei et al., 2015). For instance, Iuculano et al. (2014) found that elementary school children with high-functioning autism (HFA) demonstrated superior mathematical problem-solving abilities compared to their typically developing (TD) peers. This pattern has been attributed to ASD-related strengths in rule-based, systematic, and detail-oriented processing (Baron-Cohen et al., 2007). However, other studies adopting a deficit perspective have reported that many individuals with ASD experience significant mathematical difficulties (Bullen et al., 2020; Titeca et al., 2015a, 2015b). A meta-analysis by Chiang and Lin (2007) indicated that the majority of individuals with ASD (ages 3–51 years) show notable mathematical deficits, with the prevalence of mathematical difficulties estimated to be three to five times higher than in TD children (Jones et al., 2009; Oswald et al., 2016). Thus, whether ASD is associated with mathematical strengths or deficits may depend on the specific cognitive demands of the mathematical task.

A second perspective concerns the role of IQ. Studies that have controlled for verbal and nonverbal IQ often report reduced or absent group differences in mathematical performance (Titeca et al., 2015a, 2015b; Troyb et al., 2014). For example, Titeca et al. (2017) observed no significant differences in mathematical performance between preschool children (aged 4–5 years) with HFA and TD peers after accounting for IQ. This suggests that general cognitive ability may account for some of the observed variance in mathematical outcomes across studies.

A third perspective highlights the heterogeneity of the ASD population. Individuals with ASD vary widely in language ability, symptom severity, and co-occurring conditions, all of which may influence mathematical development. Consistent with this view, Kandel et al. (2021) noted that only 13% of individuals with HFA displayed mathematical talent, while the majority showed average or below-average performance. This heterogeneity likely contributes to divergent findings across studies, as different samples may capture different segments of the ASD spectrum.

Taken together, these theoretical perspectives suggest that the relationship between ASD and mathematical abilities is not uniform. Rather, it may depend on the type of mathematical skill assessed, the cognitive profile of the individual, and the specific sample characteristics. Thus, it remains uncertain under what conditions individuals with ASD demonstrate stronger or weaker mathematical abilities compared to their TD peers.

### 1.2. Mathematical Ability and the Acuity of ANS

Mathematical ability is influenced by a multitude of factors. These factors can be broadly categorized into two types: general domain abilities (e.g., language proficiency and intelligence) and domain-specific abilities (e.g., the acuity of the approximate number system and symbolic number knowledge) (Nunes et al., 2015; Purpura & Simms, 2018; Östergren & Träff, 2013). Research has indicated that early mathematical development in humans relies on the approximate number system (ANS). The ANS is a cognitive system present across developmental stages and species, including preverbal infants, non-human animals, and adults (Xu & Spelke, 2000; Brannon & Merritt, 2011). This innate system underpins the representation, conceptualization, and manipulation of numerical information (Halberda & Feigenson, 2008).

Evidence suggests that the acuity of the ANS serves as a cognitive foundation for later symbolic mathematical abilities (Brankaer et al., 2014). Several studies have reported a significant correlation between ANS acuity and mathematical achievement across a wide age range, even after controlling for confounding variables such as literacy skills (Halberda & Feigenson, 2008; Libertus et al., 2016). However, this relationship is not consistently observed. For instance, Kolkman et al. (2013) found no significant predictive effect of ANS acuity on the mathematical ability of 4- and 5-year-old children, a result echoed by Holloway and Ansari (2009) in their study of children aged 6 to 8 years.

The distinction between formal and informal mathematical abilities has been emphasized in research on mathematical learning difficulties (Mazzocco & Thompson, 2005) and may help explain the mixed findings regarding the association between ANS acuity and mathematical performance (Libertus et al., 2016). Yet, controversy remains over which type of mathematical ability—formal or informal—is more strongly predicted by ANS acuity. Libertus et al. (2013) conducted a longitudinal study with children aged 3 to 7 and found that ANS acuity predicted informal but not formal mathematical ability, after controlling for age and intelligence. In contrast, other studies have shown that individuals with severe impairments in formal mathematics also exhibit reduced ANS acuity (Mazzocco et al., 2011), and that ANS acuity remains correlated with formal mathematical ability in adults even when language skills are accounted for (Libertus et al., 2012).

### 1.3. The Present Study

Taken together, these theoretical perspectives lead to specific predictions. First, if ASD-related mathematical difficulties emerge with increasing demands for formal instruction, group differences may be more pronounced in older children. Second, children with ASD may rely more heavily on rule-based, ANS-mediated processing for formal mathematics. This pattern is consistent with the weak central coherence account (Happé & Frith, 2006) and the systemizing account (Baron-Cohen, 2009) of ASD. If so, then ANS acuity should be associated with formal mathematics in the ASD group but not in the TD group. The present study employed a cross-sectional design to investigate mathematical abilities and their association with the ANS in Chinese preschoolers with and without ASD. We focused on children aged 3 to 7 years. This is a critical period for the emergence of mathematical competencies (Libertus et al., 2013). We addressed the following research questions:

RQ1: Do age-related differences in formal and informal mathematical abilities exist between children with ASD and their typically developing peers?

RQ2: Is ANS acuity associated with formal and informal mathematical abilities within each group (ASD and TD)?

RQ3: Does the relationship between ANS and mathematical abilities differ between formal and informal mathematics within each group?

Based on the theoretical perspectives outlined above, we formulated the following hypotheses:

**H1:**  *No group differences in mathematical abilities are expected at ages 3–5 years, but at ages 6–7 years, children with ASD will show lower abilities than TD peers.*

**H2:**  *ANS acuity will be associated with both formal and informal mathematics in the ASD group, but only with informal mathematics in the TD group.*

**H3:**  *The ANS-formal math association will be stronger in ASD, and the ANS-informal math association will be stronger in TD.*

Specifically, we pursued three primary research aims. First, we sought to compare age-related differences in formal and informal mathematical abilities between children with ASD and their typically developing peers. Rather than inferring developmental trajectories from cross-sectional data, we examined whether group differences in mathematical performance varied across three age bands: 3–4 years, 5 years, and 6–7 years. Second, we aimed to examine the association between ANS acuity and mathematical abilities within each group. Given the heterogeneity inherent in ASD, we conducted separate analyses for the ASD and TD groups to identify group-specific predictive patterns. Third, we explored whether the relationship between ANS and mathematical abilities differed across types of mathematical abilities (formal vs. informal) within each group. Given the exploratory nature of this study and the modest sample size, we focused on descriptive patterns and within-group associations rather than formal tests of group-by-age interactions.

To address these aims, we assessed mathematical abilities using the Test of Early Mathematics Ability–Third Edition (TEMA-III; Ginsburg & Baroody, 2003), which provides separate indices of informal and formal mathematical skills. ANS acuity was measured using the Panamath dot comparison task (Halberda & Feigenson, 2008), a well-established measure for young children. Verbal IQ was assessed with the Chinese version of the Peabody Picture Vocabulary Test–Third Edition (PPVT-III), and nonverbal IQ was evaluated using Raven’s Coloured Progressive Matrices (CPM). These instruments were selected for their established reliability and validity in both typically developing children and those with ASD (L. Wang et al., 2023; Alhamdan et al., 2023; Lozano-Ruiz et al., 2021).

## 2. Methods

### 2.1. Participants

#### 2.1.1. Sample Size Consideration

Based on similar studies exploring the relationship between mathematical abilities and the ANS in children (Libertus et al., 2013; Halberda & Feigenson, 2008), an a priori power analysis was conducted using G*Power 3.1 (Faul et al., 2007) for a repeated measures ANOVA. The analysis indicated that a sample size of 60 participants per group would be required to detect a medium effect size (η^2^ = 0.06) with 80% power and α = 0.05. Given the well-documented heterogeneity of ASD and the challenges associated with recruiting this population, we initially aimed to recruit 100 participants (50 ASD, 50 TD). However, due to attrition (three children with ASD were unable to complete all assessments), the final sample consisted of 94 children aged 3 to 7 years: 47 children with ASD and 47 age- and gender-matched TD children.

#### 2.1.2. ASD Group

Children with ASD were recruited from three special education rehabilitation institutions in Haikou municipality, Hainan Province, China. The final ASD sample included 47 children (M_age_ = 69.98 months, SD = 12.63; 14 girls, 33 boys). All children in the ASD group had received a clinical diagnosis of autism spectrum disorder from qualified physicians based on DSM-5 and ICD-10 diagnostic criteria. To confirm eligibility and characterize the sample, the Childhood Autism Rating Scale (CARS; Schopler et al., 1980) was administered to all children with ASD. All participants scored above the cutoff of 30, confirming the diagnosis (range: 30–47). Based on standard CARS classification criteria—scores of 30–36 indicating mild autism and scores of 37 or above indicating moderate-to-severe autism—the sample included 32 children with mild autism and 15 children with moderate-to-severe autism.

#### 2.1.3. TD Group

The TD group consisted of 47 children recruited from a public kindergarten in the same municipality (M_age_ = 67.17 months, SD = 11.10; 14 girls, 33 boys). Children in the TD group had no reported developmental delays, psychiatric conditions, or special education needs, as confirmed by parent and teacher reports. The ASD and TD groups were matched on chronological age (*t*(92) = 1.15, *p* > .05) and gender distribution (χ^2^ = 0.00, *p* > .05).

#### 2.1.4. Age Group Stratification

Based on developmental milestones in early numerical cognition—specifically, the emergence of counting principles around age 3–4 (Brooks et al., 2013) and the integration of counting with numerical magnitude knowledge by age 6 (Jordan et al., 2010)—participants were divided into three age bands for subgroup analyses: 3–4 years (*n* = 22; 10 ASD, 12 TD; age range: 3:0–4:11), 5 years (*n* = 21; 12 ASD, 9 TD; age range: 5:0–5:11), and 6–7 years (*n* = 51; 25 ASD, 26 TD; age range: 6:0–7:11). The stratification into three age groups resulted in relatively small and uneven subsamples across age bands, which may limit statistical power for detecting age-specific effects and reduce the comparability of findings across subgroups. Therefore, results from the age-stratified analyses should be interpreted with caution.

#### 2.1.5. Ethical Considerations

This study was approved by the Ethics Committee of Hainan Normal University Prior to participation, all parents or legal guardians were informed of the study’s purpose and procedures and provided written informed consent. All children provided verbal assent prior to testing. All participants were right-handed, had no history of organic or mental illness, and had normal or corrected-to-normal vision.

#### 2.1.6. Use of AI-Assisted Language Editing

The authors used DeepSeek-V3.2 (DeepSeek Company) for language polishing, primarily in the Abstract and revised sections. All AI-generated suggestions were reviewed and verified by the authors, who take full responsibility for the manuscript.

### 2.2. Measures

#### 2.2.1. Formal and Informal Mathematical Abilities

The Test of Early Mathematics Ability III (TEMA-III) developed by Ginsburg and Baroody (2003) is suitable for children aged 3 to 8 years old. There are a total of 72 questions in this test, with 1 point for correct answers and 0 point for incorrect answers. If you answer 5 consecutive questions incorrectly, the test will be stopped. Chinese scholars have used this test to assess Chinese young children and found that it has good reliability and validity, making it an effective tool for evaluating children’s early mathematical abilities (Kang et al., 2014; L. Wang et al., 2023). The test is divided into two sub tests based on the ability requirements tested by the questions: the informal mathematical abilities test (including counting, numerical magnitude comparison, estimations, and simple numerical concepts, totaling 40 questions) and the formal mathematical abilities test (including numerical reading and writing, numerical facts, numerical operations, and numerical concepts understanding, totaling 32 questions). In this study, the Cronbach’s alpha coefficients for the informal and formal mathematics proficiency sub tests were 0.95 and 0.94, respectively, and the overall internal consistency coefficient of the test was 0.97.

#### 2.2.2. The Acuity of ANS

The Panamath children’s ANS acuity testing tool, developed and designed by Halberda and Feigenson (2008), is used for a dot matrix comparison task. The main device is a Dell laptop (resolution 1366 × 768, refresh rate 60.005 Hz). The Panamath program runs on the Java platform, first presenting a “+” fixation point on the computer screen, then pressing the space bar to simultaneously display blue and yellow dots for 2128 milliseconds. Children need to select the dot matrix with the larger number of colors based on the quantity, press the corresponding color button to make a judgment (as the participants are ASD and TD children, the button task is completed by the experimenter, and children only need to identify which side of the dot matrix has more quantities). The experimenter should make corresponding button responses based on the child’s reaction. If the child fails to respond within the presentation time, the stimulus will disappear and a masked stimulus will be presented, followed by a response box. Only after the child responds can they proceed to the next attempt. Each dot matrix consists of 5 to 21 dots, with the ratio of yellow and blue dots ranging from 1.2 to 2.8. Dot matrices of various ratios are randomly presented, and this test includes 2 practice trials and 96 experimental trials. Half of the trials had more yellow dots, while the other half had more blue dots. Half of the trials are consistent trials (lattices with the same number of points and total area size), while the other half are inconsistent trials.

#### 2.2.3. Verbal IQ

As a measure of verbal IQ in Chinese, the Peabody Picture Vocabulary Test (Chinese version) Third Edition (PPVT III) was used to evaluate children’s language development, mainly in terms of comprehension vocabulary(Zhao et al., 2021). In this study, children were instructed to listen to the experimenter and select one picture out of four that best matched the word uttered by the experimenter. If children did not respond for more than 30 s, it would be treated as an error. It consists of 125 items in the test, with a score of 1 for correct answer and 0 for incorrect answer. When a participant gave six incorrect responses out of eight consecutive items, the test came to an end. The score for this task is calculated by subtracting the number of incorrect items from the highest number of correct items answered by the child.

#### 2.2.4. Nonverbal IQ

The nonverbal IQ of participants was measured by the Raven’s Coloured Progressive Matrices (CPM) (Raven et al., 1998), which is more suitable for intelligence assessment in ASD or young children (Rindermann & Ackermann, 2021). This task consists of 36 progressively difficult items, including an incomplete pattern with six options below, of which only one is correct. The highest score for this test is 36, with a score of 1 for correct answer and 0 for incorrect answer.

## 3. Results

To address our three research questions (RQs), we first report descriptive statistics and group comparisons (RQ1). We then present age-related differences in mathematical abilities, followed by age-related differences in ANS acuity. Next, we examine the associations between ANS acuity and mathematical abilities within each group (RQ2). Finally, we test whether these associations differ between formal and informal mathematics within each group (RQ3).

### 3.1. Descriptive Statistics and Group Comparisons

Table 1 presents the means, standard deviations, and group comparisons for all study variables. Independent samples *t*-tests revealed that children with ASD scored significantly lower than TD children on informal mathematical abilities (*t*(92) = 2.31, *p* = 0.023, Cohen’s *d* = 0.48) and ANS accuracy (*t*(92) = 2.45, *p* = 0.016, Cohen’s *d* = 0.51), but not on formal mathematical abilities (*t*(92) = 0.85, *p* = 0.398) or ANS Weber fraction (*t*(92) = 1.12, *p* = 0.266).

### 3.2. Age-Related Differences in Mathematical Abilities

**Figure 1:**
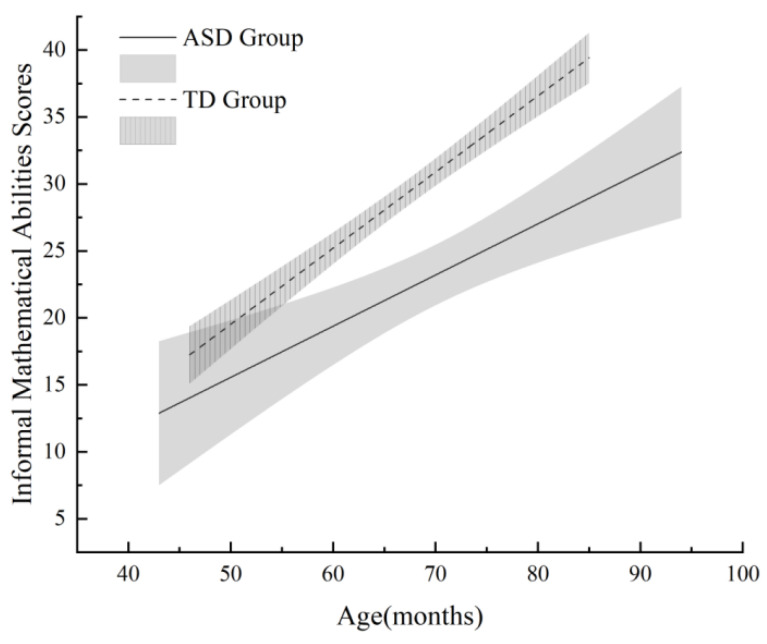
Changes in informal mathematical abilities of ASD and TD children with age (shaded areas represent 95% confidence intervals).

**Figure 2:**
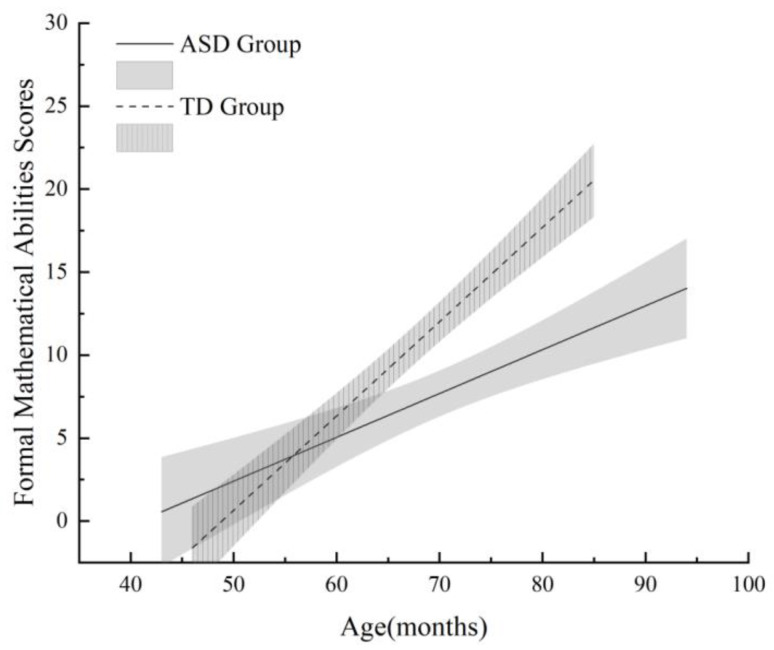
Changes in formal mathematical abilities of ASD and TD children with age (shaded areas represent 95% confidence intervals).

*Correlations with age.* Figure 1 and Figure 2 illustrate the relationships between age (in months) and mathematical abilities. Informal mathematical abilities were positively correlated with age in both the ASD group (*r*(45) = 0.54, *p* < 0.001) and the TD group (*r*(45) = 0.89, *p* < 0.001), indicating that older children in both groups achieved higher scores. Similarly, formal mathematical abilities were positively correlated with age in both the ASD group (*r*(45) = 0.58, *p* < 0.001) and the TD group (*r*(45) = 0.85, *p* < 0.001). Fisher’s *r*-to-*z* transformations confirmed that the correlations were significantly stronger in the TD group than in the ASD group for both informal (*z* = 3.21, *p* < 0.01) and formal (*z* = 2.98, *p* < 0.01) mathematical abilities, suggesting that the age-related progression of mathematical skills is more pronounced among typically developing children.

**Figure 3:**
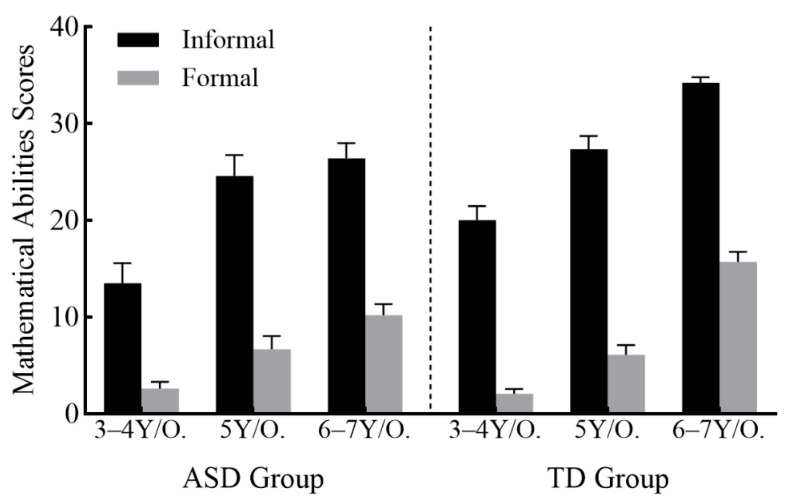
Mathematical abilities scores for ASD and TD children aged 3–7 (Error bars are se).

*Age-stratified group comparisons.* To examine whether group differences in mathematical abilities varied across developmental stages, we conducted a 2 (Mathematical Ability: informal vs. formal) × 2 (Group: ASD vs. TD) × 3 (Age Band: 3–4 years, 5 years, 6–7 years) mixed ANOVA, with mathematical ability as a within-subjects factor and group and age band as between-subjects factors. Verbal and nonverbal IQ were included as covariates to control for their potential influence on mathematical performance. Estimated marginal means are presented in Figure 3.

The analysis revealed a significant main effect of age band, *F*(2, 86) = 26.66, *p* < 0.001, $\eta_{p}^{2}$ = 0.383, indicating that older children demonstrated higher mathematical abilities than younger children. The main effect of group was not significant, *F*(1, 86) = 0.65, *p* = 0.422, $\eta_{p}^{2}$ = 0.007, suggesting no overall difference in mathematical abilities between children with and without ASD when collapsing across age.

Crucially, the Group × Age Band interaction was significant, *F*(2, 86) = 6.22, *p* = 0.003, $\eta_{p}^{2}$ = 0.126, indicating that the pattern of age-related differences varied between groups. Simple effects analyses revealed the following:

Within the ASD group: Children in the 3–4 years band scored significantly lower than those in the 6–7 years band (*p* = 0.003). However, no significant differences emerged between the 3–4 years and 5 years bands (*p* = 0.066) or between the 5 years and 6–7 years bands (*p* = 0.627).

Within the TD group: Children in both the 3–4 years and 5 years bands scored significantly lower than those in the 6–7 years band (*p*s < 0.001). The difference between the 3–4 years and 5 years bands was not significant (*p* = 0.081).

Between-group comparisons within each age band: No significant group differences emerged in the 3–4 years band (*p* = 0.603) or the 5 years band (*p* = 0.522). However, in the 6–7 years band, children with ASD scored significantly lower than their TD peers (*p* < 0.001).

*Effects involving mathematical ability type.* The main effect of mathematical ability was significant, *F*(1, 86) = 29.76, *p* < 0.001, $\eta_{p}^{2}$ = 0.257, with both groups scoring higher on informal than formal mathematical tasks. This effect was qualified by a significant Mathematical Ability × Group interaction, *F*(1, 86) = 7.29, *p* = 0.008, $\eta_{p}^{2}$ = 0.078. Simple effects analyses showed that for informal mathematical abilities, children with ASD scored marginally lower than TD children (MASD = 23.92, SE = 0.83; MTD = 26.16, SE = 0.76), *F*(1, 86) = 3.42, *p* = 0.068, $\eta_{p}^{2}$ = 0.038. For formal mathematical abilities, no significant group difference emerged (MASD = 7.98, SE = 0.73; MTD = 7.36, SE = 0.66), *F*(1, 86) = 0.34, *p* = 0.561, $\eta_{p}^{2}$ = 0.004.

The Mathematical Ability × Age Band interaction was also significant, *F*(2, 86) = 5.59, *p* = 0.005, $\eta_{p}^{2}$ = 0.115. Simple effects analyses revealed:

For informal mathematical abilities: Children in the 3–4 years band scored significantly lower than those in both the 5 years (*p* < 0.001) and 6–7 years (*p* < 0.001) bands. The difference between the 5 years and 6–7 years bands was not significant (*p* = 0.065).

For formal mathematical abilities: Children in both the 3–4 years and 5 years bands scored significantly lower than those in the 6–7 years band (*p*s < 0.001). The difference between the 3–4 years and 5 years bands was not significant (*p* = 0.373).

The three-way Mathematical Ability × Group × Age Band interaction was not significant, *F*(2, 86) = 1.96, *p* = 0.148, $\eta_{p}^{2}$ = 0.044, indicating that the two-way interactions described above were consistent across age bands. However, given our a priori interest in group differences across development, we conducted follow-up simple-simple effects analyses examining the Group × Age Band interaction separately for each type of mathematical ability (see Table 2 for full simple effects results). For informal mathematical abilities, within the ASD group, children in the 3–4 years band scored significantly lower than those in the 5 years (*p* = 0.005) and 6–7 years (*p* = 0.001) bands, with no difference between the 5 years and 6–7 years bands (*p* = 0.997). Within the TD group, all pairwise comparisons were significant: the 3–4 years band scored lower than the 5 years band (*p* = 0.047) and the 6–7 years band (*p* < 0.001), and the 5 years band scored lower than the 6–7 years band (*p* = 0.010). For formal mathematical abilities, within the ASD group, no significant pairwise differences emerged between any age bands (all *p*s > 0.066). Within the TD group, both the 3–4 years and 5 years bands scored significantly lower than the 6–7 years band (*p*s < 0.001), but the difference between the 3–4 years and 5 years bands was not significant (*p* = 0.393).

### 3.3. Age-Related Differences in ANS Acuity

In order to investigate the developmental differences in ANS acuity between children with ASD and TD in three age groups, a 2 (child type: ASD, TD) × 3 (age group: 3–4 years old, 5 years old, 6–7 years old) MANOVA was again conducted with ANS acuity (accuracy and Weber fraction) as the two dependent variables, and child type and age group as the independent variables. To control for the impact of general cognitive abilities (such as language proficiency and intelligence) on the acuity of ANS, we used verbal and nonverbal IQ as covariates in the analysis of variance. The results showed that the main effect of child type on ANS acuity was significant, with *F*(1, 86) = 10.30, *p* = 0.002, $\eta_{p}^{2}$ = 0.107, indicating that the accuracy of ANS in children with ASD is significantly lower than that in children with TD. However, the main effect of child type on the ANS Weber fraction is not significant, with *F*(1, 86) = 2.93, *p* = 0.090, $\eta_{p}^{2}$ = 0.033. The main effects of age group on ANS accuracy [*F*(2, 86) = 0.97, *p* = 0.384, $\eta_{p}^{2}$ = 0.022] and ANS Weber fraction [*F*(2, 86) = 1.79, *p* = 0.174, $\eta_{p}^{2}$ = 0.040] were not significant. And there was no significant two-way child type × age group interaction in terms of ANS accuracy [*F*(2, 86) = 0.06, *p* = 0.941, $\eta_{p}^{2}$ = 0.001] and ANS Weber fraction [*F*(2, 86) = 0.13, *p* = 0.882, $\eta_{p}^{2}$ = 0.003].

To examine whether ANS acuity differed between groups and across age bands, we conducted a 2 (Group: ASD vs. TD) × 3 (Age Band: 3–4 years, 5 years, 6–7 years) multivariate analysis of covariance (MANCOVA), with ANS accuracy and ANS Weber fraction as dependent variables. Verbal and nonverbal IQ were included as covariates to control for their potential influence on ANS performance.

The analysis revealed a significant multivariate main effect of group, Wilks’Λ = 0.89, *F*(2, 85) = 5.21, *p* = 0.007, $\eta_{p}^{2}$ = 0.109. Follow-up univariate analyses indicated that this effect was driven by ANS accuracy: children with ASD demonstrated significantly lower accuracy than their TD peers (MASD = 0.72, SE = 0.03; MTD = 0.81, SE = 0.03), *F*(1, 86) = 10.30, *p* = 0.002, $\eta_{p}^{2}$ = 0.107. However, for the ANS Weber fraction, the group difference did not reach statistical significance (MASD = 0.28, SE = 0.02; MTD = 0.24, SE = 0.02), *F*(1, 86) = 2.93, *p* = 0.090, $\eta_{p}^{2}$ = 0.033.

The multivariate main effect of age band was not significant, Wilks’Λ = 0.96, *F*(4, 170) = 0.98, *p* = 0.422, $\eta_{p}^{2}$ = 0.022, indicating that ANS acuity did not differ significantly across the three age bands when collapsing across groups. Univariate analyses confirmed this pattern for both ANS accuracy, *F*(2, 86) = 0.97, *p* = 0.384, $\eta_{p}^{2}$ = 0.022, and ANS Weber fraction, *F*(2, 86) = 1.79, *p* = 0.174, $\eta_{p}^{2}$ = 0.040.

The multivariate Group × Age Band interaction was also not significant, Wilks’Λ = 0.99, *F*(4, 170) = 0.10, *p* = 0.982, $\eta_{p}^{2}$ = 0.002, suggesting that the pattern of group differences in ANS acuity was consistent across age bands. Univariate tests confirmed the absence of significant interactions for both ANS accuracy, *F*(2, 86) = 0.06, *p* = 0.941, $\eta_{p}^{2}$ = 0.001, and ANS Weber fraction, *F*(2, 86) = 0.13, *p* = 0.882, $\eta_{p}^{2}$ = 0.003.

Taken together, these results indicate that while children with ASD show lower ANS accuracy than TD children, this group difference does not vary by age, and the two groups do not differ significantly on the ANS Weber fraction. There was a discrepancy between the two ANS metrics. Specifically, we found significant group differences in accuracy but not in Weber fraction. This issue is addressed further in the Discussion.

### 3.4. Relationships Between ANS Acuity and Mathematical Abilities

*Partial correlations.* To examine the associations between ANS acuity and mathematical abilities within each group, we conducted partial correlations controlling for verbal and nonverbal IQ. Given that the two ANS metrics (accuracy and Weber fraction) and the two mathematical ability measures (informal and formal) yielded four correlations of primary interest within each group, we applied a Bonferroni correction to control for multiple comparisons, setting the adjusted alpha level at *p* < 0.0125 (0.05/4). Full correlation matrices are presented in Table 2.

As shown in Table 2, a distinct pattern emerged between the two groups. For children with ASD, both ANS accuracy and ANS Weber fraction were significantly correlated with both informal and formal mathematical abilities (all *p*s < 0.0125). Children with higher ANS accuracy and lower Weber fractions (indicating better ANS acuity) demonstrated higher scores on both types of mathematical tasks. In contrast, for TD children, ANS accuracy and ANS Weber fraction were significantly correlated with informal mathematical abilities only (*p*s < 0.0125); neither ANS metric was significantly correlated with formal mathematical abilities (*p*s > 0.05).

*Hierarchical regression analyses.* To further examine the unique contribution of ANS acuity to mathematical abilities beyond the effects of age and general cognitive abilities, we conducted a series of hierarchical regression analyses separately for the ASD and TD groups. For each analysis, mathematical ability (informal or formal) was entered as the dependent variable. In Step 1, we entered age (in months) to account for developmental effects. In Step 2, we entered verbal and nonverbal IQ as measures of general cognitive ability. In Step 3, we entered ANS accuracy and ANS Weber fraction to assess their incremental contribution.

The key pattern emerging from these analyses was that ANS acuity significantly predicted formal mathematical abilities in the ASD group, but predicted informal mathematical abilities in the TD group. Specifically, in the ASD group, ANS acuity accounted for unique variance in formal mathematics beyond age and IQ; in the TD group, ANS acuity accounted for unique variance in informal mathematics. Results are summarized in Table 3.

*Predicting mathematical abilities in children with ASD.* For informal mathematical abilities in the ASD group, age entered in Step 1 accounted for 27.1% of the variance, *F*(1, 45) = 18.09, *p* < 0.001. The addition of verbal and nonverbal IQ in Step 2 explained an additional 42.2% of the variance, Δ*F*(2, 43) = 31.07, *p* < 0.001, Δ*R*^2^ = 0.422. However, the entry of ANS accuracy and Weber fraction in Step 3 did not significantly improve the model, Δ*F*(2, 41) = 2.19, *p* = 0.125, Δ*R*^2^ = 0.019, indicating that ANS acuity does not make a unique contribution to informal mathematical abilities in children with ASD beyond age and IQ.

For formal mathematical abilities in the ASD group, age entered in Step 1 accounted for 33.8% of the variance, *F*(1, 45) = 22.98, *p* < 0.001. The addition of verbal and nonverbal IQ in Step 2 explained an additional 30.2% of the variance,

Δ*F*(2, 43) = 18.00, *p* < 0.001, Δ*R*^2^ = 0.302. Critically, the entry of ANS accuracy and Weber fraction in Step 3 explained an additional 5.4% of unique variance, and this increment was statistically significant, Δ*F*(2, 41) = 3.64, *p* = 0.035, Δ*R*^2^ = 0.054. Examination of the final model revealed that ANS accuracy was a significant unique predictor (β = 0.28, *p* = 0.042), while ANS Weber fraction was not (β = −0.15, *p* = 0.263).

*Predicting mathematical abilities in children with TD.* For informal mathematical abilities in the TD group, age entered in Step 1 accounted for a substantial 78.3% of the variance, *F*(1, 45) = 162.38, *p* < 0.001. The addition of verbal and nonverbal IQ in Step 2 did not significantly improve the model, Δ*F*(2, 43) = 0.48, *p* = 0.624, Δ*R*^2^ = 0.004. However, the entry of ANS accuracy and Weber fraction in Step 3 explained an additional 5.4% of unique variance, and this increment was statistically significant, Δ*F*(2, 41) = 7.01, *p* = 0.002, Δ*R*^2^ = 0.054. In the final model, both ANS accuracy (β = 0.31, *p* = 0.008) and ANS Weber fraction (β = −0.24, *p* = 0.031) emerged as significant unique predictors.

For formal mathematical abilities in the TD group, age entered in Step 1 accounted for 72.2% of the variance, *F*(1, 45) = 116.70, *p* < 0.001. The addition of verbal and nonverbal IQ in Step 2 did not significantly improve the model, Δ*F*(2, 43) = 0.63, *p* = 0.536, Δ*R*^2^ = 0.006. The entry of ANS accuracy and Weber fraction in Step 3 also failed to reach statistical significance, Δ*F*(2, 41) = 2.88, *p* = 0.068, Δ*R*^2^ = 0.022, although the increment approached significance. Neither ANS accuracy (β = 0.19, *p* = 0.112) nor ANS Weber fraction (β = −0.16, *p* = 0.178) was a significant unique predictor in the final model.

*Summary of regression findings.* The regression analyses revealed a dissociation between the two groups. For children with ASD, ANS acuity made a unique contribution to formal mathematical abilities only (5.4% unique variance), not to informal abilities. For TD children, ANS acuity made a unique contribution to informal mathematical abilities only (5.4% unique variance), not to formal abilities. Notably, in both instances where ANS contributed significantly, it accounted for exactly 5.4% of the unique variance, suggesting a modest but reliable effect. The theoretical and practical implications of this dissociation are addressed in the Discussion.

## 4. Discussion

### 4.1. Age-Related Differences in Mathematical Abilities

The present study examined mathematical abilities in Chinese preschoolers with and without ASD, revealing both similarities and differences in how these abilities manifest across age. Three main findings emerged regarding the age-related patterns of mathematical abilities in this population. (We use “age-related patterns” rather than “development” because our data are cross-sectional.)

First, consistent with previous research (Titeca et al., 2014), we found that children with ASD demonstrated significant age-related improvements in both informal and formal mathematical abilities. Older children in both groups outperformed younger children, showing age-related differences in mathematical competencies regardless of diagnostic status. This finding aligns with studies showing that basic numerical competencies are present in children with ASD and differ across age (Bullen et al., 2020; Titeca et al., 2015a).

However, the pattern of age-related differences diverged from some previous reports. For instance, L. Wang et al. (2023) found that young children with ASD aged 3–6 years performed significantly below TD peers and showed a different pattern of age-related differences. The discrepancy between our findings and those of Wang et al. may be attributable to differences in sample characteristics. In the Wang et al. study, the ASD and TD groups differed substantially in both verbal and nonverbal intelligence (Cohen’s *d*s = 4.91 and 3.64, respectively), whereas in the present study, the groups were well-matched on IQ (Cohen’s *d* = −0.31). Given the well-established association between general cognitive abilities and mathematical performance (Bullen et al., 2020), the more pronounced deficits observed in Wang et al.’s ASD sample may reflect their lower cognitive functioning rather than ASD-specific mathematical difficulties per se. This highlights the importance of carefully matching or controlling for cognitive abilities when comparing mathematical competencies across groups.

Second, we observed that both groups scored significantly higher on informal than formal mathematical abilities. This finding is consistent with the typical pattern of mathematical cognition more generally. Informal mathematical knowledge is acquired through everyday experiences such as counting objects or comparing quantities, and it typically precedes and forms the foundation for formal, symbol-based mathematical skills taught in school (Ginsburg & Baroody, 2003; Raman, 2002). For Chinese preschoolers, who are predominantly exposed to informal, play-based mathematical activities in kindergartens and rehabilitation settings, formal mathematical problems involving written symbols and abstract operations may pose greater challenges (Cheng et al., 2019). This pattern held for both children with and without ASD, suggesting that the informal-to-formal progression is a general feature of early mathematical development rather than a phenomenon specific to either group.

Third, and most critically, we found that the magnitude of group differences varied across age. While no significant differences emerged between children with ASD and TD in the 3–4 years and 5 years age bands, a significant gap appeared in the oldest age band (6–7 years), with children with ASD underperforming relative to their TD peers. This pattern was particularly pronounced for informal mathematical abilities, where children with ASD showed consistently lower scores than TD children across all age bands, and the gap widened at older ages. For formal mathematical abilities, the group difference at 6–7 years appeared to reflect a widening gap rather than a consistent lag: children with ASD showed similar levels of formal mathematical ability between ages 5 and 7, whereas TD children showed higher levels at age 7 compared to age 5.

The emergence of group differences only in the oldest age band may reflect the changing nature of mathematical demands as children approach school entry. Before age 5, mathematical competencies are predominantly informal and embedded in everyday contexts—counting objects, comparing quantities, and basic numerical concepts that can be acquired through naturalistic interaction with the environment (Sun, 2003). Children with ASD may leverage their strengths in rote memory and procedural learning to perform adequately on these tasks (Kim & Cameron, 2016). However, as children transition to formal schooling around age 6–7, mathematical tasks increasingly require symbolic representation, abstract reasoning, and integration of multiple concepts. These skills draw more heavily on language and cognitive flexibility, areas where many children with ASD experience challenges (Titeca et al., 2014, 2017).

This interpretation aligns with Titeca and colleagues’ finding that children with ASD perform similarly to TD peers on early number processing tasks at age 5–6, but show significant deficits by age 6–7 on tasks requiring math fact retrieval and written problem-solving (Titeca et al., 2014). Similarly, Kim and Cameron (2016) noted that children with ASD may excel at memorizing arithmetic facts but struggle with more complex skills such as solving word problems or equations. The present findings extend this work by demonstrating that this pattern holds in a Chinese sample and emerges specifically during the transition to formal schooling.

Importantly, we interpret these findings as reflecting age-related differences rather than developmental trajectories per se. Because our data are cross-sectional, we cannot determine whether the observed patterns reflect true developmental change or cohort effects. Longitudinal studies following the same children across the preschool-to-school transition are needed to confirm whether the gap that emerges at age 6–7 represents a genuine divergence in developmental pathways. What our findings do suggest is that by age 6–7, children with ASD are already lagging behind their peers in mathematical competencies, underscoring the importance of early monitoring and intervention.

### 4.2. Acuity of ANS in Children with ASD

The present study found that children with ASD showed significantly lower ANS acuity than their TD peers, primarily reflected in lower accuracy on the dot comparison task. This finding is consistent with previous research documenting ANS deficits in young children with ASD (L. Wang et al., 2023; X. Li et al., 2024).

One possible explanation for this difficulty relates to the cognitive demands of the ANS task itself. The numerical magnitude comparison task requires children to attend to a set of quantities (the dot array) as a whole rather than focusing on individual dots. Successful performance depends on extracting global numerical information while resisting the tendency to focus on local perceptual features. According to the Weak Central Coherence (WCC) theory, individuals with ASD tend to process information locally rather than globally, showing a preference for detailed features of stimuli and weaker integration of information into coherent wholes (Burnette et al., 2005; Happé & Frith, 2006). This local processing bias may place children with ASD at a disadvantage when judging the relative numerosity of dot arrays, as they may be more likely to focus on individual dot properties (e.g., size, position) rather than the overall numerical magnitude (Aagten-Murphy et al., 2015).

An additional consideration is the potential bidirectional relationship between ANS acuity and formal mathematics learning. While the ANS is present from infancy and is thought to provide a foundation for the acquisition of symbolic mathematics (Brankaer et al., 2014; Feigenson et al., 2004; Libertus et al., 2013), emerging evidence suggests that formal mathematics education may also shape ANS acuity over time (Piazza et al., 2013; Lindskog et al., 2014). Longitudinal studies have reported reciprocal associations between symbolic mathematics knowledge and ANS acuity, suggesting that each may be related to the other across development (Elliott et al., 2019; Mou et al., 2023).

This bidirectional perspective could inform how we interpret ANS-related findings in children with ASD, although causal directions remain to be tested. Compared to their TD peers, many children with ASD have limited access to formal mathematics instruction in mainstream educational settings. Even when children with ASD receive intervention services in rehabilitation institutions, mathematics education is often not a primary focus (Sullivan & Wang, 2020). Thus, rather than viewing lower ANS acuity in ASD as purely a core deficit or purely a consequence of reduced mathematics exposure, it may be more accurate to conceptualize it as arising from a combination of factors: atypical perceptual processing tendencies (consistent with WCC) that affect initial ANS task performance, coupled with reduced opportunities for mathematics learning that might otherwise help refine ANS acuity through reciprocal feedback mechanisms. Future longitudinal research is needed to disentangle the direction and nature of these relationships.

### 4.3. The Relationship Between Mathematical Abilities and ANS Acuity in Children with ASD

A substantial body of research, including both cross-sectional (e.g., Keller & Libertus, 2015; J. J. Wang et al., 2016) and longitudinal studies (e.g., Libertus et al., 2013; Malone et al., 2019), has documented significant associations between preschool children’s mathematical abilities and their ANS acuity. The present study extends this work by examining these relationships separately in children with and without ASD, revealing distinct patterns of association between the two groups.

*Patterns in typically developing children.* For TD children, we found that ANS acuity was significantly correlated with and predicted informal mathematical abilities, but showed no significant association with formal mathematical abilities. This pattern aligns closely with findings from Libertus et al. (2013). They assessed ANS acuity in 85 children aged 3–7 years on four occasions over two years. After controlling for age and intelligence, they found that ANS acuity was significantly related to informal but not formal mathematical abilities as measured by the TEMA-III. Libertus and colleagues suggested that ANS may support the acquisition of foundational numerical competencies, such as mastering the verbal counting system and understanding ordered relationships between numerical symbols. At the same time, the ANS may play a less direct role in mastering formal mathematical conventions like place value systems and basic fact retrieval. Our findings in TD children replicate this pattern in an independent sample, further supporting the view that the ANS–formal math link may be indirect or mediated by other cognitive skills in typically developing populations.

*Patterns in children with ASD*. A different pattern emerged for children with ASD. In this group, ANS acuity was significantly correlated with both informal and formal mathematical abilities, and hierarchical regression analyses revealed that ANS made a unique contribution to formal mathematical abilities specifically, accounting for 5.4% of the variance after controlling for age and IQ. This is a modest but statistically significant effect size (Cohen, 2013). This finding suggests that the relationship between ANS and mathematical competencies may operate differently in children with ASD compared to their TD peers. From a practical standpoint, this modest effect implies that while ANS acuity is not the sole determinant of formal mathematics in ASD, it does explain a non-trivial portion of individual differences beyond age and IQ.

One possible explanation for this difference relates to the lower ANS acuity observed in the ASD group. Consistent with previous research on children with mathematical learning disabilities (Mazzocco et al., 2011), we found that children with ASD showed impaired ANS performance relative to TD children. When ANS acuity is compromised, its relationship with symbolic mathematics may become more pronounced. According to the mapping account proposed by Holloway and Ansari (2009), the association between ANS and formal mathematical abilities may be mediated by the precision with which children can map non-symbolic ANS representations onto formal numerical symbols. For children with intact ANS acuity, this mapping process may operate efficiently and thus show weaker direct correlations with formal math outcomes, as other factors (e.g., instruction, language, memory) come to play larger roles. However, when ANS acuity is poor, the underlying non-symbolic representations are imprecise. This may constrain the development of symbolic numerical knowledge, resulting in a stronger observable relationship between ANS and formal mathematics. This pattern was seen in the ASD group in the present study.

One possible, though speculative, explanation is that the relationship between ANS and formal mathematics is bidirectional and could be related to differential mathematics learning experiences, though this interpretation remains speculative given our cross-sectional data. Children with ASD in our sample may have had fewer opportunities for formal mathematics instruction. This pattern, also reported in previous studies (Sullivan & Wang, 2020), could limit the feedback effects that formal mathematics learning typically exerts on ANS refinement (Piazza et al., 2013; Elliott et al., 2019). If ANS acuity is not being sharpened through formal mathematics engagement, its baseline level may remain a more consistent predictor of mathematical outcomes. However, longitudinal data are needed to examine this bidirectional hypothesis.

It is important to note that these interpretations are tentative. The cross-sectional design of the present study precludes conclusions about directionality or causation. Moreover, the modest effect size (5.4% unique variance) indicates that while ANS makes a statistically significant contribution to formal mathematics in ASD, a substantial portion of variance remains unexplained by ANS, age, and IQ combined. Future longitudinal research with larger samples is needed to determine whether the observed patterns reflect stable group differences in how ANS supports mathematical learning. Additional factors that may moderate these relationships, such as executive functions, language abilities, and educational experiences, should also be examined in future studies.

## 5. Limitations and Future Directions

Due to the cross-sectional design of this study, no causal interpretations can be drawn from our findings. All reported associations are correlational. Several limitations of the present study should be acknowledged, which also point to important directions for future research.

*Sample size and statistical power*. First, although we aimed to recruit 50 children per group based on a priori power analysis, the final sample of 47 children with ASD and 47 TD children fell slightly short of this target. More importantly, the stratification of participants into three age bands (3–4 years, 5 years, and 6–7 years) resulted in relatively small subsamples within each age band (ranging from 21 to 51 participants, with some subgroups as small as 9–12 children per cell). This limited statistical power has important implications for interpretation. First, low statistical power increases the risk of Type II errors—that is, failing to detect true group differences or associations, particularly for interaction effects such as Group × Age Band. Second, small and uneven subsamples may lead to unstable estimates of effect sizes, reducing the reliability and generalizability of the findings. This limited statistical power also precluded more complex analyses such as formal tests of Group × Age interactions. The uneven sample sizes across age bands, especially the larger 6–7 years group, further complicate age-related comparisons. Therefore, the results involving age-stratified comparisons and interaction effects should be interpreted with caution. Future studies with larger and more balanced samples are needed to replicate the age-stratified findings and to examine whether the patterns observed here hold across development.

*Heterogeneity within the ASD group*. Second, children with ASD are known to exhibit considerable heterogeneity in cognitive profiles and symptom severity. While we reported CARS scores to characterize the sample (32 children with mild autism, 15 with moderate-to-severe autism), the modest sample size prevented us from systematically examining whether symptom severity moderates the relationship between ANS acuity and mathematical abilities. Previous research suggests that cognitive and academic outcomes may differ substantially across the autism spectrum (H. H. Li et al., 2020). Future studies with larger samples should investigate whether the ANS-math link varies as a function of ASD severity or subtype, as this could inform more targeted intervention strategies.

*Uncontrolled family and environmental factors*. Third, this study did not account for family-level variables that previous research has consistently linked to early mathematical development, including parental education, socioeconomic status, home numeracy environment, and parental involvement in mathematical activities. These factors may differ systematically between families of children with and without ASD and could partially account for the observed group differences. In addition, access to formal instruction (e.g., preschool enrollment, curriculum quality, and early educational interventions) may also vary across children and influence mathematical development. Future research should include comprehensive measures of the home learning environment to disentangle child-level cognitive factors from environmental influences.

*Measurement of mathematical abilities*. Fourth, while we followed the TEMA-III framework in distinguishing between informal and formal mathematical abilities, each of these broad categories encompasses multiple specific competencies (e.g., counting, number comparison, estimation, arithmetic operations, place value understanding). The developmental trajectories of these specific skills may differ both within and between groups. Future research employing more fine-grained measures of mathematical subdomains could provide a more comprehensive understanding of the mathematical development of children with ASD.

*Cross-sectional design*. Fifth, and most fundamentally, the cross-sectional design of this study limits the conclusions that can be drawn. As noted throughout the discussion, our findings reflect age-related differences rather than developmental trajectories per se. We cannot determine whether the patterns observed, especially the emergence of group differences at age 6–7, reflect true developmental change or cohort effects. Moreover, the cross-sectional design precludes causal inferences about the direction of the relationship between ANS acuity and mathematical abilities. While we have interpreted the findings in terms of ANS potentially supporting mathematical learning, bidirectional relationships are plausible, with formal mathematics instruction also shaping ANS acuity over time (Piazza et al., 2013; Elliott et al., 2019). Longitudinal studies tracking the same children across the preschool-to-school transition are urgently needed to establish the developmental course of these skills and to test causal hypotheses about their interrelations.

*Statistical considerations*. Finally, although we found that ANS acuity accounted for a significant 5.4% of unique variance in formal mathematical abilities in the ASD group, this effect size is modest. The majority of variance remains unexplained by the variables included in our models. Moreover, our analytical approach, which involved conducting separate regressions for each group, does not provide a direct statistical test of whether the predictive strength of ANS differs significantly between groups. Future studies with larger samples should employ moderated regression analyses to formally test Group × ANS interactions, which would provide a more rigorous basis for claiming group differences in predictive patterns.

Despite these limitations, the present study makes an important contribution by providing the first systematic comparison of ANS-math relationships in Chinese preschoolers with and without ASD. The findings suggest that the role of ANS in mathematical development may differ between these populations, with implications for early identification and intervention. By acknowledging these limitations and outlining directions for future research, we hope to stimulate further investigation into this important but understudied area.

## 6. Conclusions

The present cross-sectional study examined mathematical abilities and their relationship with the ANS in Chinese preschoolers with and without ASD. Three main findings emerged. First, while children with ASD and their TD peers performed similarly on mathematical tasks at ages 3–4 and 5 years, a significant gap emerged by ages 6–7 years, with children with ASD underperforming relative to TD children—a pattern particularly pronounced for informal mathematical abilities. Second, distinct patterns of association between ANS acuity and mathematical abilities were observed: for TD children, ANS was correlated with informal mathematical abilities only, whereas for children with ASD, ANS was correlated with both informal and formal abilities and made a unique contribution to formal mathematical abilities, accounting for 5.4% of the variance after controlling for age and IQ. Third, children with ASD showed significantly lower ANS accuracy than their TD peers, consistent with accounts of local processing biases that may interfere with global magnitude processing. Together, these findings suggest that the relationship between foundational number sense and mathematical competencies differs between preschoolers with and without ASD, and that by school entry, children with ASD may already lag behind their peers in mathematical readiness. Early intervention programs for preschoolers with ASD should consider incorporating activities that target non-symbolic magnitude comparison skills, though given the modest effect sizes and cross-sectional design, these recommendations remain tentative and warrant confirmation through longitudinal research.

## Figures and Tables

**Table 1:** Descriptive statistics of all research variables in children with ASD and TD (mean ± SD).

Variables	ASD Group (*n* = 47)	TD Group (*n* = 47)	Min.	Max.	*t*	Cohen’s *d*
Age (months)	69.98 ± 12.63	67.17 ± 11.10	43	94	1.15	0.24
MA	30.87 ± 14.15	39.68 ± 14.13	8	66	−3.02 **	−0.62
Informal MA	23.19 ± 9.01	29.28 ± 7.13	7	40	−3.63 ***	−0.75
Formal MA	7.68 ± 5.74	10.40 ± 7.42	0	26	−1.99 *	−0.41
ANS Accuracy	81.99 ± 14.06	95.88 ± 5.19	51.92	100	−6.36 ***	−1.31
ANS Weber fraction	0.60 ± 0.73	0.14 ± 0.12	0.02	3.24	4.29 ***	0.88
Verbal IQ	52.65 ± 22.74	76.17 ± 16.86	16	105	−5.70 ***	−1.17
Nonverbal IQ	22.67 ± 7.25	24.57 ± 4.56	6	36	−1.52	−0.31

Note: * *p* < 0.05, ** *p* < 0.01, *** *p* < 0.001; MA: Mathematical abilities; ANS: Approximate Number System.

**Table 2:** Partial correlations after controlling for age differences among all the test scores.

	1	2	3	4	5	6	7
1 Verbal IQ	—	0.25	0.26	−0.15	0.05	−0.08	−0.02
2 Nonverbal IQ	0.38	—	0.20	−0.16	0.15	0.12	0.15
3 ANS Accuracy	0.46 *	0.51 *	—	−0.90 *	0.45 *	0.27	0.40
4 ANS Weber fraction	−0.42	−0.56 *	−0.89 *	—	−0.51 *	−0.34	−0.48 *
5 Informal MA	0.57 *	0.69 *	0.60 *	−0.55 *	—	0.54 *	0.86 *
6 Formal MA	0.52 *	0.60 *	0.61 *	−0.54 *	0.76 *	—	0.90 *
7 MA	0.59 *	0.70 *	0.64 *	−0.58 *	0.97 *	0.90 *	—

Note: * *p* < 0.05, Bonferroni-corrected; MA: Mathematical abilities; ANS: Approximate Number System; The correlation coefficient for TD children is in the upper right corner of the diagonal, while the correlation coefficient for ASD children is in the lower left corner of the diagonal.

**Table 3:** Hierarchical regression analyses predicting mathematical abilities in children with ASD and TD.

Group	Dependent Variable	Step	Predictors	Δ*R*^2^	Δ*F*	*p*
ASD	Informal Math	1	Age	0.271	18.09	<0.001
		2	Verbal IQ, Nonverbal IQ	0.422	31.07	<0.001
		3	ANS Accuracy, ANS Weber	0.019	2.19	0.125
	Formal Math	1	Age	0.338	22.98	<0.001
		2	Verbal IQ, Nonverbal IQ	0.302	18.00	<0.001
		3	ANS Accuracy, ANS Weber	0.054	3.64	**0.035**
TD	Informal Math	1	Age	0.783	162.38	<0.001
		2	Verbal IQ, Nonverbal IQ	0.004	0.48	0.624
		3	ANS Accuracy, ANS Weber	0.054	7.01	**0.002**
	Formal Math	1	Age	0.722	116.70	<0.001
		2	Verbal IQ, Nonverbal IQ	0.006	0.63	0.536
		3	ANS Accuracy, ANS Weber	0.022	2.88	0.068

Note. Δ*R*^2^ = *R*^2^ change at each step. Bold indicates statistically significant increments (*p* < 0.05).

## Data Availability

The original contributions presented in this study are included in the article. Further inquiries can be directed to the corresponding author.
